# Difference of central foveal thickness measurement in patients with macular edema using optical coherence tomography in different display modes

**DOI:** 10.3389/fendo.2024.1295745

**Published:** 2024-01-26

**Authors:** Dan Jouma Amadou Maman Lawali, Guanrong Wu, Nouhou Diori Adam, Zhangjie Lin, Huiqian Kong, Liaohui Yi, Ying Fang, Yongyi Niu, Changting Tang, Abdou Amza, Hongyang Zhang, Honghua Yu, Ling Yuan, Yijun Hu

**Affiliations:** ^1^Guangdong Eye Institute, Department of Ophthalmology, Guangdong Provincial People’s Hospital (Guangdong Academy of Medical Sciences), Southern Medical University, Guangzhou, China; ^2^Department of Ophthalmology, Amirou Boubacar Diallo National Hospital, Abdou Moumouni University of Niamey, Niamey, Niger; ^3^Affiliated Hospital of Guilin Medical University, Guilin, China; ^4^Guangdong Provincial Key Laboratory of Artificial Intelligence in Medical Image Analysis and Application, Guangzhou, China; ^5^Department of Ophthalmology, The First Affiliated Hospital of Kunming Medical University, Kunming, China

**Keywords:** optical coherence tomography, optical coherence tomography image tilting angle, central foveal thickness, display mode, macular edema

## Abstract

**Purpose:**

To assess the differences in the measurement of central foveal thickness (CFT) in patients with macular edema (ME) between two display modes (1:1 pixel and 1:1 micron) on optical coherence tomography (OCT).

**Design:**

This is a retrospective, cross-sectional study.

**Methods:**

Group A consisted of participants with well-horizontal OCT B-scan images and group B consisted of participants with tilted OCT B-scan. We manually measured the CFT under the two display modes, and the values were compared statistically using the paired *t*-test. Spearman’s test was used to assess the correlations between the OCT image tilting angle (OCT ITA) and the differences in CFT measurement. The area under the curve (AUC) was calculated to define the OCT ITA cutoff for a defined CFT difference.

**Results:**

In group A, the mean CFT in the 1:1 pixel display mode was 420.21 ± 130.61 µm, similar to the mean CFT of 415.27 ± 129.85 µm in the 1:1 micron display mode. In group B, the median CFT in the 1:1 pixel display mode is 409.00 μm (IQR: 171.75 μm) and 368.00 μm (IQR: 149.00 μm) in the 1:1 micron display mode. There were significant differences between the two display modes with the median (IQR) absolute difference and median (IQR) relative difference of 38.00 μm (75.00 μm) and 10.19% (21.91%) (all *p* = 0.01). The differences in CFT measurement between the two display modes were correlated with the OCT ITA (absolute differences, *r* = 0.88, *p* < 0.01; relative differences, *r* = 0.87, *p* < 0.01). The AUC for a predefined CFT difference was 0.878 (10 μm), 0.933 (20 μm), 0.938 (30 μm), 0.961 (40 μm), 0.962 (50 μm), and 0.970 (60 μm).

**Conclusion:**

In patients with DM, when the OCT B-scan images were well-horizontal, manual CFT measurements under the two display modes were similar, but when the B-scan images were tilted, the CFT measurements were different under the two display modes, and the differences were correlated to the OCT ITA.

## Introduction

Macular edema (ME) is one of the major causes of vision impairment in patients suffering from metabolic, vascular, and inflammatory retinal disorders (1–5). The etiology of ME includes diabetes, retinal vein occlusion (RVO), epiretinal membrane (ERM), and age-related macular degeneration (AMD) (3, 6–11). ME affects approximately 7 million people worldwide due to diabetes and approximately 3 million people due to venous occlusions. In developed countries, neovascular age-related macular degeneration represents 5% of ME among subjects over the age of 60 (12).

Spectral-domain optical coherence tomography (SD-OCT) is a non-invasive and standard diagnostic tool for detecting ME, as well as for monitoring and follow-up after treatment. As a result, SD-OCT has a considerable influence on decisions about the management of ME, particularly in common disorders such as DME and AMD (13–16). It allows the measurement of individual retinal layers, the determination of retinal thickness and macular volume, and the qualitative assessment of fluid distribution. Even though this was done manually before, several recent studies employing machine learning aim to automate the quantification of fluid and other distinctive factors (10, 14, 17). Although central macular thickness (CMT) measurements automatically evaluate the average retinal thickness within a 1-mm concentric circle (14, 18–20), clinicians are still using SD-OCT’s central foveal thickness (CFT) manual measurements to assess ME, which represents a fundamental marker for diagnosing ME in different retinal diseases (21–23).

It is known that there are two display modes on OCT: the 1:1 pixel display mode and the 1:1 micron display mode. The 1:1 pixel display mode represents the most commonly used display mode for OCT images in daily clinical practice. The ratio between vertical and horizontal scales is 3.775 in the 1:1 pixel display mode and 1.0 in the 1:1 micron display mode. Previously, we found significant differences in CFT measurements between the two OCT display modes in myopic patients, and the differences were correlated to the OCT image tilting angle (OCT ITA) (24). However, whether our findings can be applied to patients with ME is yet to be confirmed.

The aim of the current study was to evaluate the differences in manual measurements of the CFT under the two display modes (1:1 pixel display mode and 1:1 micron display mode) in patients with ME. In addition, we investigated the OCT ITA cutoff for some predefined CFT differences between the two OCT display modes.

## Materials and methods

### Participants

In this retrospective, cross-sectional study, we recruited subjects with ME treatment-naive and post-treatment of different origins who visited the Ophthalmology Department of Guangdong Provincial People’s Hospital (GDPH) and who received the SD-OCT scanning. The subjects’ characteristics included age, gender, lens status eyes with ME, BCVA between 0.01 and 1.0 logMAR, and CFT more than 300 μm on SD-OCT.

We excluded eyes with severe cataracts, IOP >21 mmHg, and eyes that could not be scanned using SD-OCT due to poor patient cooperation. All subjects underwent comprehensive ophthalmic examinations, including best-corrected visual acuity with a decimal chart, slit-lamp biomicroscope examination of the anterior segment and the fundus, intraocular pressure, and SD-OCT scanning.

This study was approved by the Institutional Review Board (IRB) of GDPH and followed the Helsinki Declaration. Furthermore, the necessity for informed consent was waived by the same IRB because no specific subject can be identified from the data.

### Imaging

Before SD-OCT scanning, 0.5% tropicamide (Santen Pharmaceutical Co., Ltd. Shiga Plant) was used to achieve adequate pupillary dilation. Participants were directed to look at the machine’s fixation light, and the foveolar fixation was confirmed by visualizing the retinal image through the infrared monitoring camera.

**Figure 1:**
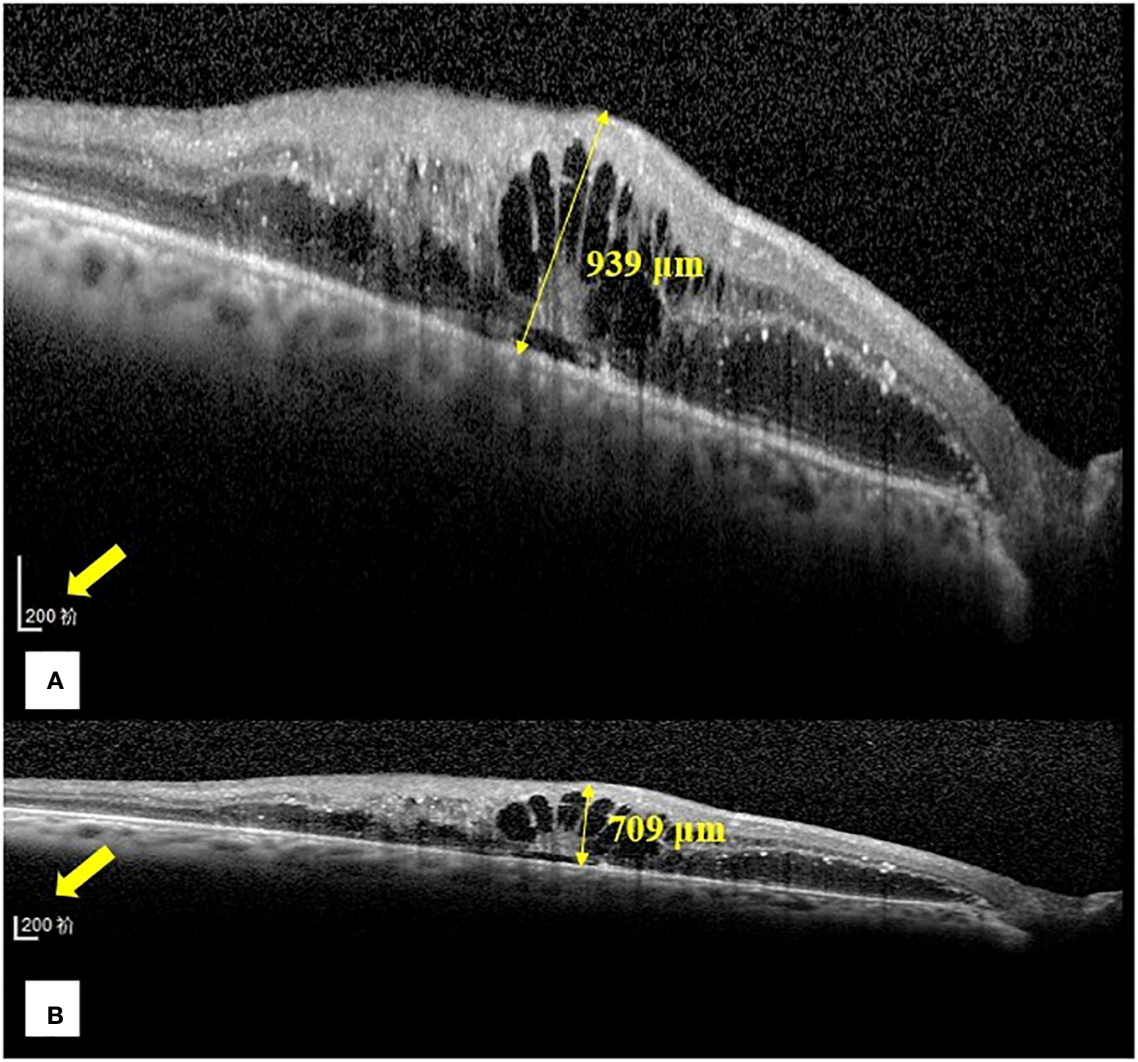
Representative images of the central foveal thickness manual measurement in the two optical coherence tomography display modes (1:1 pixel display mode and 1:1 micron display mode) in group **(B)**. **(A)** Central foveal thickness manual measurement in the 1:1 pixel display mode. **(B)** Central foveal thickness manual measurement in the 1:1 micron display mode. In the 1:1 pixel display mode, the central foveal thickness is 939 μm, and in the 1:1 micron display mode, it is 709 μm.

**Figure 2:**
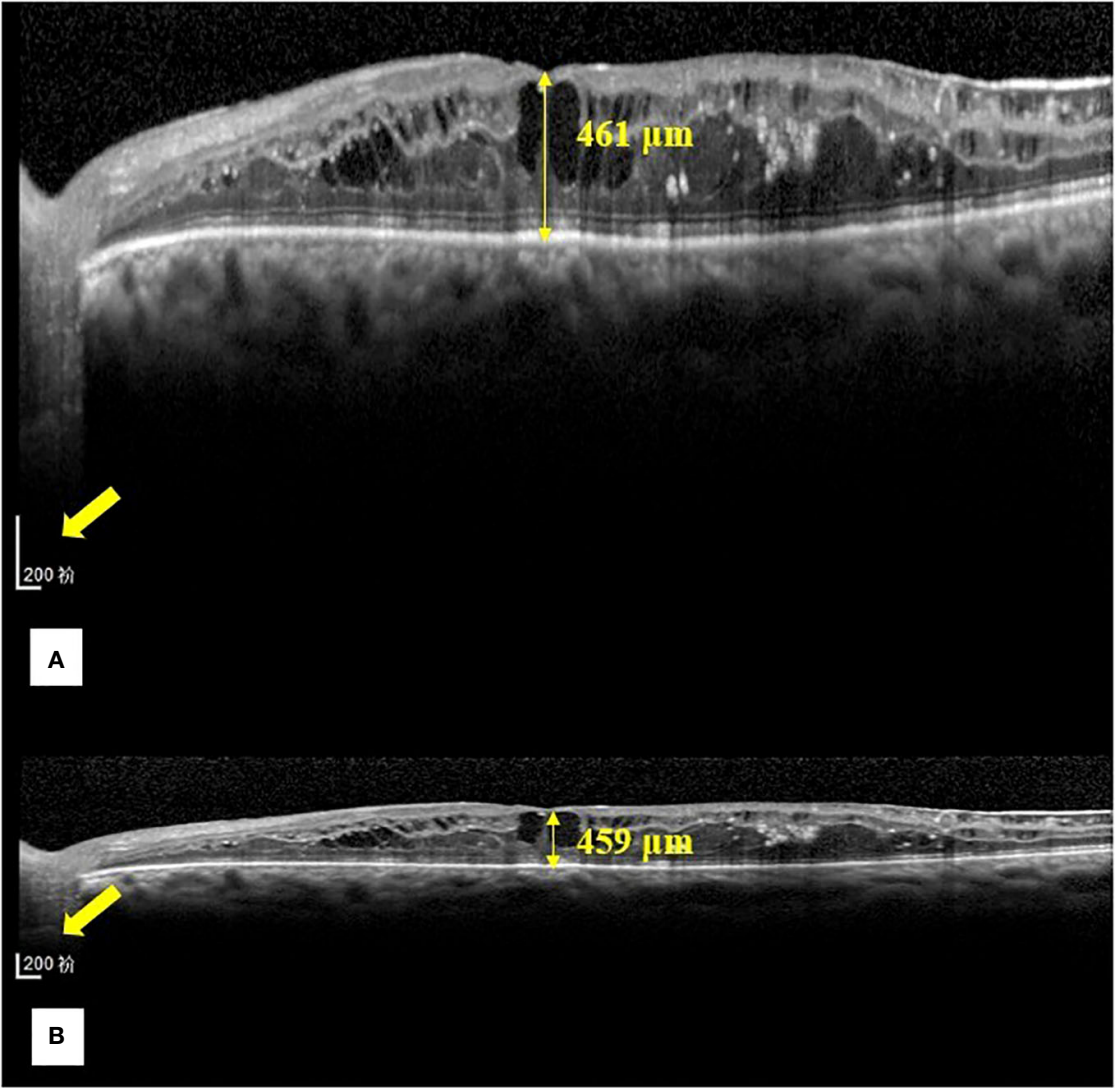
Representative images of the central foveal thickness manual measurement in the two OCT display modes (1:1 pixel display mode and 1:1 micron display mode) in group **(A)**. **(A)** Central foveal thickness manual measurement in the 1:1 pixel display mode. **(B)** Central foveal thickness manual measurement in the 1:1 micron mode. In the 1:1 pixel display mode, the central foveal thickness is 461 μm, and in the 1:1 micron display mode, it is 459 μm.

We performed a high-resolution horizontal B-scan for each eye through the central fovea using SD-OCT (Spectralis, Heidelberg Engineering GmbH, Heidelberg, Germany), and an OCT image of the right eye was selected for manual measurement. If the right eye image was not accessible, the left eye image was used. We divided the participants into two groups. Group A included patients with well-horizontal OCT B-scan images, and group B included patients with tilted OCT B-scan images. Manual measurement was accomplished by the same experienced ophthalmologist (D.M.). All measurements were conducted in both display modes, i.e., 1:1 pixel display mode and 1:1 micron display mode (**Figures 1**, **2**). SD-OCT’s caliper measuring tool was used to manually measure the CFT in the two display modes.

**Figure 3:**
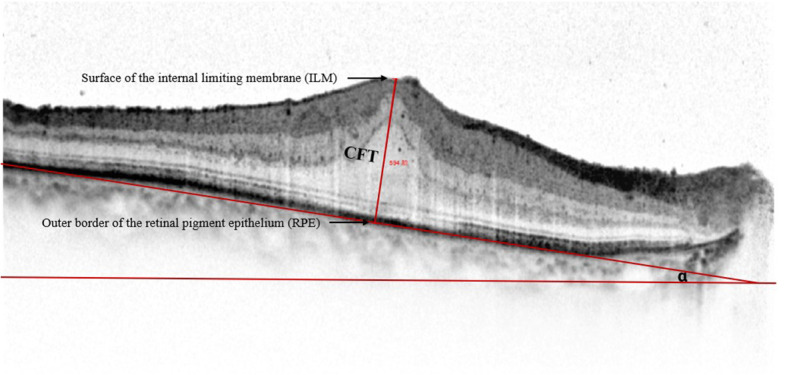
Representative spectral-domain optical coherence tomography image of the central foveal thickness and the optical coherence tomography image tilting angle. Central foveal thickness is defined as the distance between the surface of the internal limiting membrane and the outer border of the retinal pigment epithelium at the foveal zone. Optical coherence tomography image tilting angle is the angle alpha between the line parallel to the edge of the optical coherence tomography image and the line tangent to the retinal pigment epithelium.

We defined CFT as the vertical distance between the surface of the internal limiting membrane and the outer border of the retinal pigment epithelium (RPE) (**Figure 3**). We used the ImageJ software (US National Institutes of Health, Bethesda, USA; https://imagej.net/software/fiji/) to assess the OCT ITA. To measure the OCT ITA, we first drew a line tangent to the RPE line below the foveola. The angle between this line and the bottom edge of the OCT image was designated as the OCT ITA (**Figure 3**).

### Statistical analysis

The data are presented as mean and standard deviation (mean ± SD) for the data not normally distributed; otherwise, they are presented as median and interquartile range (IQR). The statistical analyses were performed using SPSS 26.0 (SPSS. Inc., Chicago, IL, USA). The Shapiro–Wilk test was used to assess the normality of the data. The OCT measurement in the two display modes was compared using the paired *t*-test. The absolute difference in CFT measurement was calculated by CFT in 1:1 pixel mode minus CFT in 1:1 micron mode, and the relative difference in CFT measurement was defined as the absolute difference divided by the retinal thickness in 1:1 micron mode. Spearman correlation tests and linear regressions were used to assess the correlations between the OCT ITA and the differences in OCT measurement under the two display modes. For all the tests, *p <*0.05 was considered statistically significant.

## Results

### Baseline demographic characteristics and underlying pathologies of ME in the two groups

**Table 1** shows the baseline demographic characteristics and the underlying pathologies of ME in group A and group B. We recruited 52 participants in group A, consisting of 29 (55.8%) patients with DME, 3 (5.8%) patients with RVO, 19 (36.5%) patients with ERM, and 1 (1.9%) patient with AMD, with a mean age of 63.15 ± 12.13 years, lens status ratio (phakic/pseudophakic) of 42/10, and mean BCVA of 0.43 ± 0.32. We recruited 112 participants in group B, consisting of 40 (35.5%) patients with DME, 14 (12.5%) patients with RVO, 54 (48.2%) patients with ERM, and 4 (3.6%) patients with AMD, with a median age of 66.00 years (IQR: 15.00 years), lens status ratio of 74/38, and median BCVA of 0.30 (IQR: 0.40).

**Table 1:** Baseline demographic characteristics and the underlying pathologies of macular edema in group A and group B.

Characteristics*	Group A	Group B
Age of the patients (years)	63.15 ± 12.13	66.00 (15.00)
Lens status (phakic/pseudophakic)	42/10	74/38
BCVA	0.43 ± 0.32	0.30 (0.40)
Etiology		
DME	29 (55.8%)	40 (35.7%)
RVO	3 (5.8%)	14 (12.5%)
ERM	19 (36.5%)	54 (48.2%)
AMD	1 (1.9%)	4 (3.6%)
Total number of eyes	52 (100%)	112 (100%)

Best-corrected visual acuity (BCVA) measurement at the logarithm of the minimum angle of resolution (logMAR) scale.

*The data are statistically normally distributed. The data are presented as mean and standard deviation for the data not normally distributed; otherwise, they are presented as median and interquartile range.

**The data are presented as numbers and the percentage of eyes.

### Evaluation of the differences in CFT manual measurement between the two display modes

The evaluation of the differences (absolute and relative) between the two display modes in CFT manual measurement in group A and group B is shown in **Table 2**. In group A, the differences between the two display modes in CFT measurement are not statistically significant, with a mean CFT of 420.21 ± 130.61 µm in the 1:1 pixel display mode and a mean CFT of 415.27 ± 129.85 µm in the 1:1 micron display mode. The mean differences (absolute and relative) were 4.94 ± 4.14 µm and 1.23% ± 0.83% with *p >*0.05. In group B, we found statistically significant differences between the two display modes in the CFT manual measurement, with a median (IQR) in the 1:1 pixel display mode of 409.00 µm (171.75 µm) and 368.00 µm (149.00 µm) in the 1:1 micron display mode and a median (IQR) absolute and relative difference of 38.00 µm (75.00 µm) and 10.19% (21.91%) (all *p* < 0.05).

**Table 2:** Evaluation of the differences (absolute and relative) between the two display modes in group A as well as in group B.

CFT*	1:1 pixel (µm)	1:1 micron (µm)	Differences (absolute and relative)	*p*-value
Group A	420.21 ± 130.61	415.27 ± 129.85	4.94 μm ± 4.14 μm^†^ 1.23% ± 0.85%^††^	>0.05
Group B	409.00 (171.75)	368.00 (149.00)	38.00 μm (75.00 μm)^†^ 10.19% (21.91%)^††^	≤0.01

^†^Indicates the absolute difference.

^††^Indicates the relative difference.

*The data are statistically normally distributed. The data are presented as mean and standard deviation for the data not normally distributed; otherwise, they are presented as median and interquartile range.

### Correlations between the CFT differences in the two display modes and the OCT ITA

**Table 3** shows the correlations between the CFT differences in the two display modes and the OCT ITA. The differences in the CFT manual measurement between the two display modes were significantly correlated with the OCT ITA (absolute differences, *r* = 0.88, *p* < 0.01; relative differences, *r* = 0.87, *p* < 0.01).

**Table 3:** Correlation coefficients between the absolute and relative differences in the two OCT display modes and the OCT image tilting angle.

Differences	Angle (*r*)	*p*-value
ΔCFT (μm)*	0.88	≤0.01
ΔCFT (%)*	0.87	≤0.01

*The data are statistically not normally distributed.

**Spearman’s test was performed to predict the correlation between the absolute and the relative differences and the mean angle of measurement with r representing the correlation coefficient. Significant if p <0.05.

### The OCT ITA cutoff for a predefined CFT difference and regression equations to correct the CFT differences

**Figure 4:**
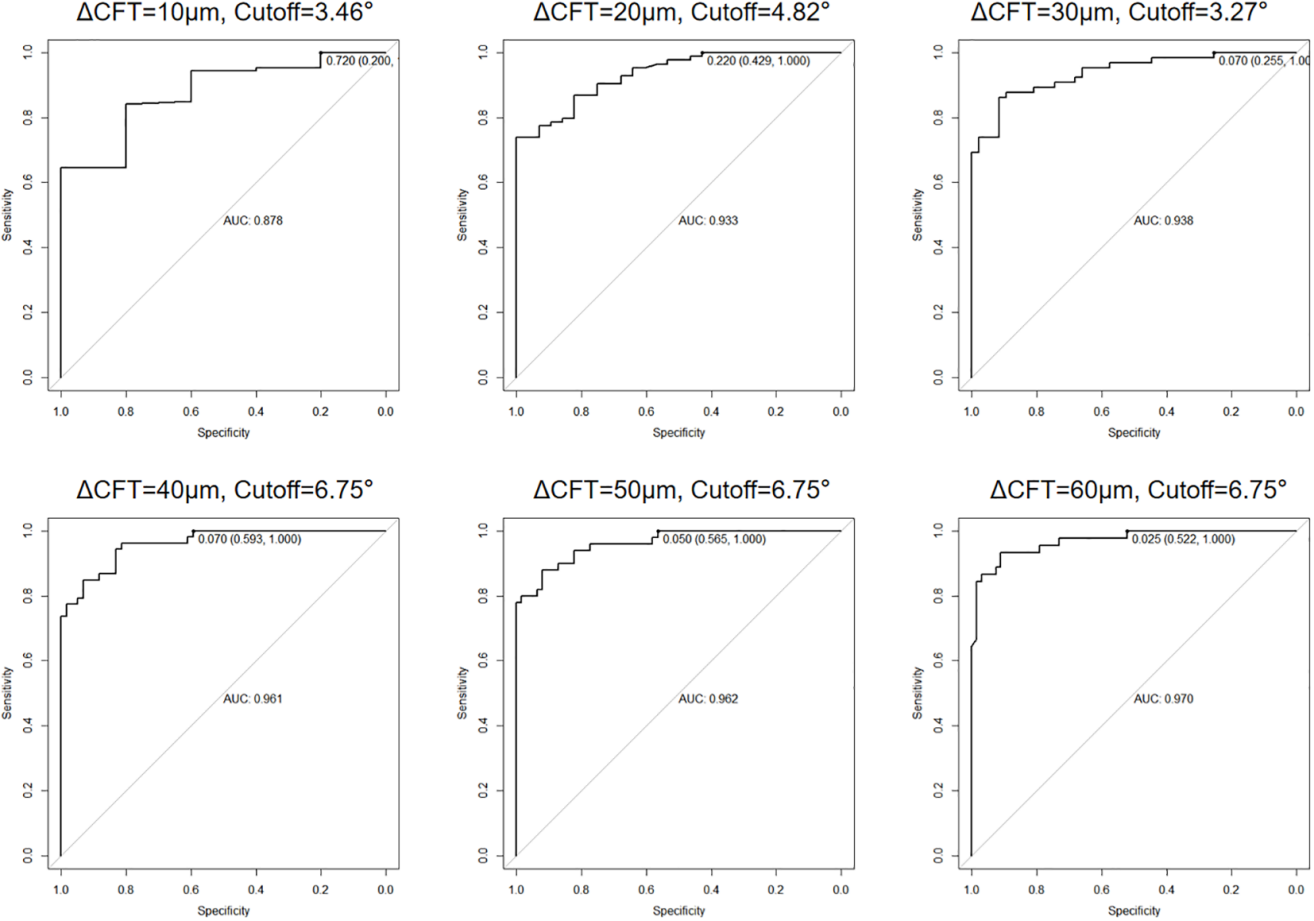
Area under the receiver operating characteristic (ROC) curve (AUC) and optical coherence tomography image tilting angle (OCT ITA) cutoff for a predefined central foveal thickness difference. As a result, the AUC of OCT ITA for a predefined central foveal thickness difference was 0.878 (10 μm), 0.933 (20 μm), 0.938 (30 μm), 0.961 (40 μm), 0.962 (50 μm), and 0.970 (60 μm).

Since the CFT differences between the two display modes were correlated to the OCT ITA, we further analyzed the OCT ITA cutoff for a predefined CFT difference using a receiver operating characteristic (ROC) curve. As shown in **Figure 4**, the area under the curve (AUC) of OCT ITA for a predefined CFT difference was 0.878 (10 μm), 0.933 (20 μm), 0.938 (30 μm), 0.961 (40 μm), 0.962 (50 μm), and 0.970 (60 μm).

**Figure 5:**
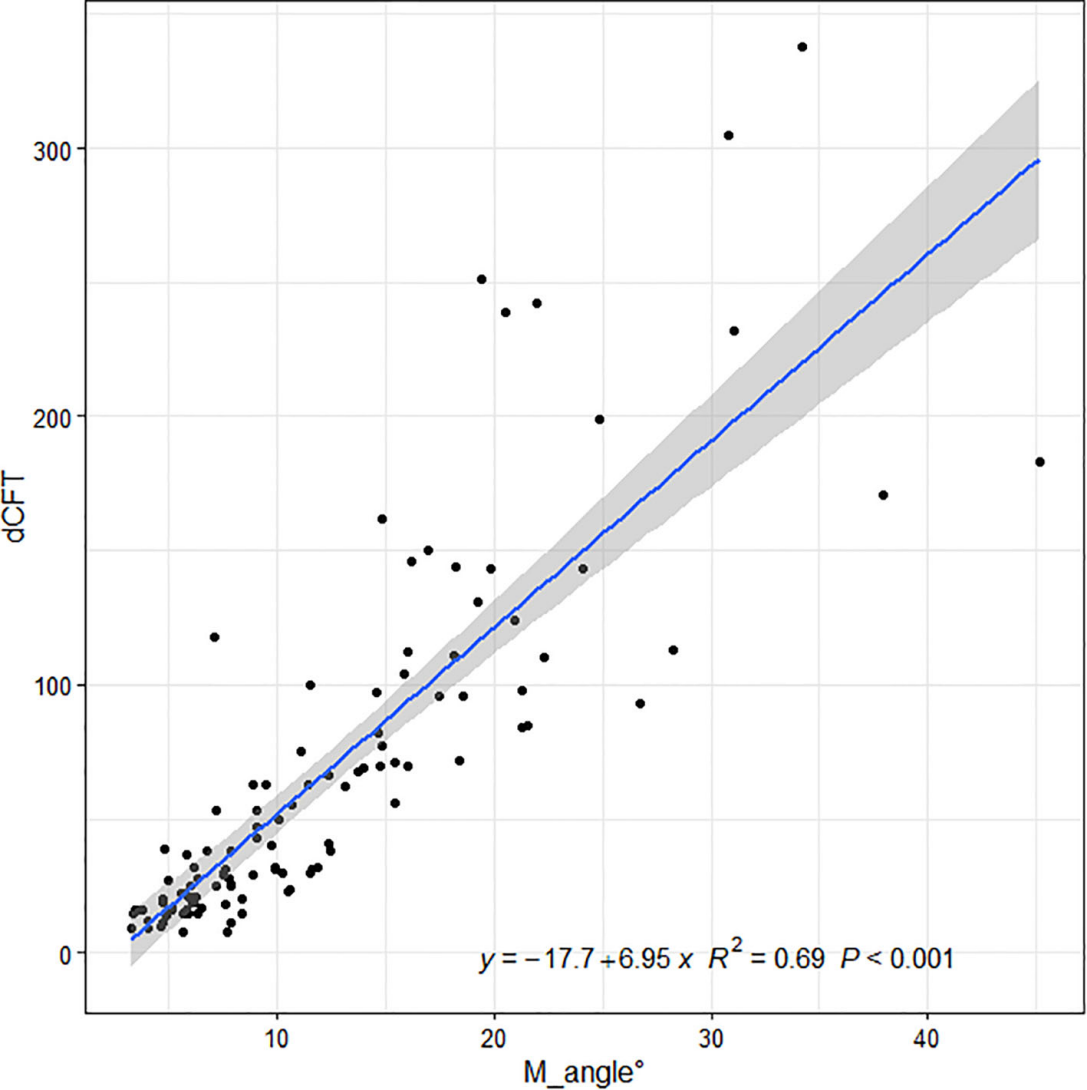
Linear regressions of the optical coherence tomography image tilting angle and the absolute differences in central foveal thickness manual measurement between the two display modes along with the regression equations. As a result, the regression equation of the optical coherence tomography image tilting angle and the absolute difference in central foveal thickness manual measurement are Y1 = −17.7 + 6.95*x* (*R*^2 = ^0.69, *p* < 0.001).

**Figure 6:**
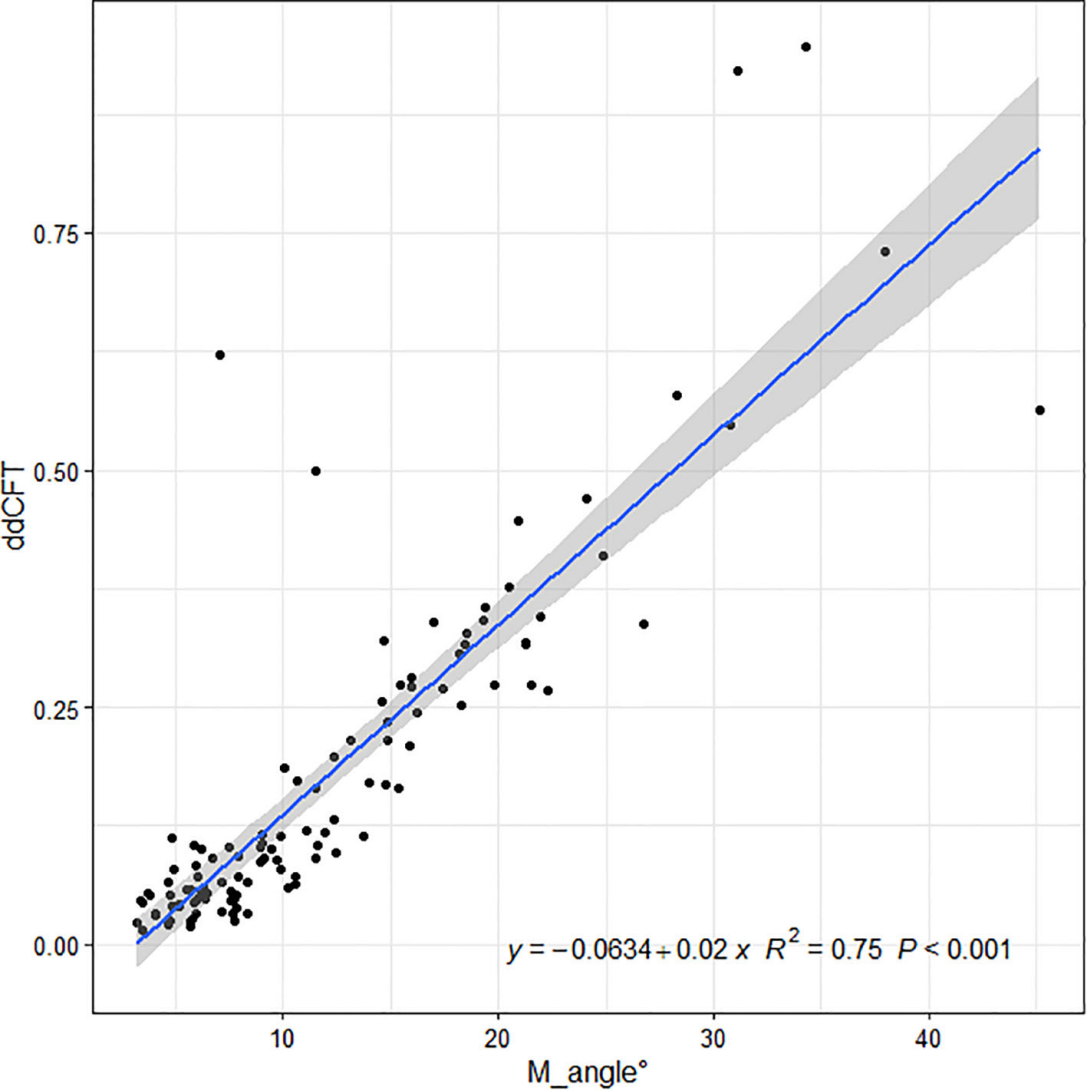
Linear regressions of the optical coherence tomography image tilting and the relative differences in central foveal thickness manual measurement between the two display modes along with the regression equations. As a result, the regression equation of the optical coherence tomography image tilting angle and the relative difference in central foveal thickness manual measurement are Y2 = −0.0634 + 0.02*x* (*R*^2 = ^0.75, *p* < 0.001).

Linear regressions of the OCT ITA and the differences in CFT measurement between the two display modes are shown in **Figures 5**, **6** along with the regression equations. The regression equations of the OCT ITA are Y1 = −17.7 + 6.95*x* (*R*^2 = ^0.69, *p* < 0.001) to correct the absolute CFT differences and Y2 = −0.0634 + 0.02*x* (*R*^2 = ^0.75, *p* < 0.001) to correct the relative CFT differences.

## Discussion

In the present study, we found significant differences in the manual measurement of CFT in patients with ME under the two OCT display modes when the OCT B-scan image is tilted. In addition, we found that the differences were correlated to the OCT ITA. To avoid measurement errors and misinterpretations of OCT B-scan images, it is beneficial to consider the differences between the two OCT display modes.

Kim et al. found differences between the two display modes for choroidal thickness measurement, and the subfoveal choroidal thickness (SFCT) was overestimated when it was measured in 1:1 pixel display mode. Consequently, they suggested that choroidal thickness should be estimated according to the 1:1 micron display mode, mainly when the estimation line is not vertical. Furthermore, they concluded that a similar measurement error can arise when measuring the thickness of other structures when the OCT B-scan image is tilted, as demonstrated in some eyes for foveal retinal thickness, which is exactly the case in our current study. However, Kim et al. did not determine the specific cause of the tilted images, but they suspected eye curvature, particularly in the case of myopia, poor fixation, head tilt, or tilt OCT camera (18). Consistently, in our previous study, we demonstrated in myopic patients that the CFT was different between the two display modes, and the differences were correlated to the OCT ITA (24).

Cho et al. found that 1:1 micron images had slightly higher interobserver measurement errors for SFCT and that choroidal thickness measurements should be evaluated with caution, particularly for a thick choroid. The SFCT measurement did not differ substantially among the observers in their study for both image display modes (*p* = 0.5663 for the 1:1 pixel mode and *p* = 0.2839 for the 1:1 micron mode, respectively). However, their investigation revealed substantially poorer repeatability from the thick CT group for the 1:1 pixel images (0.747) than the 1:1 micron images (0.75) (25). Although Kim et al. observed an overestimation in 1:1 pixel images, these errors did not appear to have a significant impact on the reproducibility (18).

While the 1:1 pixel mode displays OCT images in a ratio of 3.775 between the vertical and horizontal scales, the 1:1 micron mode displays the OCT images in a ratio of 1.0. As a result, the 1:1 micron display mode must be vertically compressed approximately three-fold in the resolution of the retinal layers. Even though the 1:1 pixel display mode can more clearly depict a precise structural change, caution should be taken when the OCT B-scan image is titled. In our study, the absolute and the relative differences between the two display modes in group B represented, respectively, 38.00 μm (75.00 μm) and 10.19% (21.91%). According to previous studies, the interobserver repeatability of manual CFT measurement is 10% (26, 27). The differences in CFT manual measurement in tilted OCT B-scan images are more significant than the interobserver repeatability stated in the previous studies. Therefore, it is necessary to correct such differences in clinical practice. Moreover, we also provided the solution for when to correct the CFT differences. The AUC was 0.878 to 0.970, depending on the cutoff value of the CFT difference used. The sensitivity of the AUC calculated was 100% for all cutoff values, indicating that it is a highly accurate tool to predict when to correct the differences. The specificity of the AUC ranged from 20.0% to 59.3%, depending on the cutoff value used, and the cutoff OCT ITA ranged from 3.27 to 6.75. These results are consistent to predict when exactly to correct the differences between the two OCT display modes.

In patients with ME, the size of macular cysts is correlated to disease severity and treatment outcomes (28–30). Thus, many clinicians use OCT manual measurement to measure the size of macular cysts in clinical practice (31–33). It is important to accurately measure the size of macular cysts for assessing ME severity and the effectiveness of treatment (30, 34). Furthermore, accurate measurement of the cyst size can also help to identify the need for further investigation, such as fluorescein angiography, which can offer more detailed information about the cyst and the underlying pathology (30, 35). According to the results of our study, it is also important to pay attention to the tilted OCT B-scan images when manually measuring the size of macular cysts because the size of the cysts may be overestimated in the 1:1 pixel mode if the OCT B-scan images are tilted.

In the case of well-horizontal OCT B-scan images, there was no significant difference in CFT measurements between the two display modes. Therefore, it is unnecessary to correct the CFT difference in these cases, and it is important for the technicians to bear in mind that well-horizontal OCT B-scan images are obtained during OCT examinations.

In our current study, after demonstrating the differences between the two OCT display modes in patients with ME and their correlation with the OCT ITA, we proposed regression equations with the OCT ITA cutoff to correct the differences because neither the product’s manufacturer nor previous literature recommended a scale of measurement.

## Conclusion

Our study found that when the OCT B-scan images were well-horizontal, manual CFT measurements in ME patients were similar under the two display modes. However, when the B-scan images were tilted, the CFT measurements in patients with ME differed under the two display modes, and these differences were correlated to the OCT ITA. We were able to correct the discrepancies between the two display modes by using the regression equations.

## Data availability statement

The raw data supporting the conclusions of this article will be made available by the authors, without undue reservation.

## Ethics statement

The studies involving humans were approved by Institutional Review Board (IRB) of GDPH and followed the Helsinki Declaration. The studies were conducted in accordance with the local legislation and institutional requirements. Written informed consent for participation was not required from the participants or the participants’ legal guardians/next of kin in accordance with the national legislation and institutional requirements.

## Author contributions

DL: Conceptualization, Data curation, Formal analysis, Investigation, Methodology, Resources, Software, Supervision, Validation, Writing – original draft, Writing – review & editing. GW: Data curation, Formal analysis, Methodology, Software, Validation, Writing – review & editing. NA: Investigation, Methodology, Supervision, Validation, Writing – review & editing. ZL: Formal analysis, Investigation, Methodology, Supervision, Validation, Writing – original draft, Writing – review & editing. HK: Validation, Writing – review & editing. LY: Data curation, Methodology, Validation, Writing – review & editing. YF: Data curation, Methodology, Validation, Writing – review & editing. YN: Conceptualization, Investigation, Methodology, Validation, Writing – review & editing. CT: Investigation, Methodology, Validation, Writing – review & editing. AA: Investigation, Methodology, Supervision, Validation, Writing – review & editing. HZ: Conceptualization, Investigation, Methodology, Validation, Writing – review & editing. HY: Conceptualization, Data curation, Formal analysis, Funding acquisition, Methodology, Project administration, Resources, Supervision, Validation, Visualization, Writing – review & editing. LY: Conceptualization, Formal analysis, Investigation, Methodology, Supervision, Validation, Visualization, Writing – original draft, Writing – review & editing. YH: Conceptualization, Data curation, Formal analysis, Investigation, Methodology, Project administration, Resources, Software, Supervision, Validation, Visualization, Writing – original draft, Writing – review & editing.

## References

[1] Yau JW Rogers SL Kawasaki R Lamoureux EL Kowalski JW Bek T. Global prevalence and major risk factors of diabetic retinopathy. Diabetes Care (2012) 35(3):556–64. doi: 10.2337/dc11-1909 PMC3322721 22301125

[2] Wang Z Zhong Y Yao M Ma Y Zhang W Li C. Automated segmentation of macular edema for the diagnosis of ocular disease using deep learning method. Sci Rep (2021) 11(1):13392. doi: 10.1038/s41598-021-92458-8 34183684 PMC8238965

[3] Forrester JV Kuffova L Delibegovic M. The role of inflammation in diabetic retinopathy. Front Immunol (2020) 11:583687. doi: 10.3389/fimmu.2020.583687 33240272 PMC7677305

[4] Bae JH Al-Khersan H Yannuzzi NA Hasanreisoglu M Androudi S Albini TA. Surgical therapy for macular edema: What we have learned through the decades. Ocul Immunol Inflamm (2019) 27(8):1242–50. doi: 10.1080/09273948.2019.1672194 31647684

[5] Tang F Qin X Lu J Song P Li M Ma X. Optical coherence tomography predictors of short-term visual acuity in eyes with macular edema secondary to retinal vein occlusion treated with intravitreal conbercept. Retina (2020) 40(4):773–85. doi: 10.1097/iae.0000000000002444 30640282

[6] Grzybowski A Markeviciute A Zemaitiene R. Treatment of macular edema in vascular retinal diseases: A 2021 update. J Clin Med (2021) 10(22):5300. doi: 10.3390/jcm10225300 34830582 PMC8619917

[7] Costa JV Moura-Coelho N Abreu AC Neves P Ornelas M Furtado MJ. Macular edema secondary to retinal vein occlusion in a real-life setting: a multicenter, nationwide, 3-year follow-up study. Graefes Arch Clin Exp Ophthalmol (2021) 259(2):343–50. doi: 10.1007/s00417-020-04932-0 32965652

[8] Das A McGuire PG Rangasamy S. Diabetic macular edema: Pathophysiology and novel therapeutic targets. Ophthalmology (2015) 122(7):1375–94. doi: 10.1016/j.ophtha.2015.03.024 25935789

[9] Rittiphairoj T Mir TA Li T Virgili G. Intravitreal steroids for macular edema in diabetes. Cochrane Database Syst Rev (2020) 11(11):Cd005656. doi: 10.1002/14651858.CD005656.pub3 33206392 PMC8095060

[10] Zur D Iglicki M Busch C Invernizzi A Mariussi M Loewenstein A. OCT biomarkers as functional outcome predictors in diabetic macular edema treated with dexamethasone implant. Ophthalmology (2018) 125(2):267–75. doi: 10.1016/j.ophtha.2017.08.031 28935399

[11] Johnson MW. Etiology and treatment of macular edema. Am J Ophthalmol (2009) 147(1):11–21.e1. doi: 10.1016/j.ajo.2008.07.024 18789796

[12] Daruich A Matet A Moulin A Kowalczuk L Nicolas M Sellam A. Mechanisms of macular edema: Beyond the surface. Prog Retin Eye Res (2018) 63:20–68. doi: 10.1016/j.preteyeres.2017.10.006 29126927

[13] de Azevedo AGB Takitani G Godoy BR Marianelli BF Saraiva V Tavares IM. Impact of manual correction over automated segmentation of spectral domain optical coherence tomography. Int J Retina Vitreous (2020) 6:4. doi: 10.1186/s40942-020-0207-6 32082615 PMC7020356

[14] Dysli M Rückert R Munk MR. Differentiation of underlying pathologies of macular edema using spectral domain optical coherence tomography (SD-OCT). Ocul Immunol Inflamm (2019) 27(3):474–83. doi: 10.1080/09273948.2019.1603313 31184556

[15] Mowatt G Hernández R Castillo M Lois N Elders A Fraser C. Optical coherence tomography for the diagnosis, monitoring and guiding of treatment for neovascular age-related macular degeneration: a systematic review and economic evaluation. Health Technol Assess (2014) 18(69):1–254. doi: 10.3310/hta18690 PMC4780940 25436855

[16] Wolf S Wolf-Schnurrbusch U. Spectral-domain optical coherence tomography use in macular diseases: a review. Ophthalmologica (2010) 224(6):333–40. doi: 10.1159/000313814 20453539

[17] Yiu G Welch RJ Wang Y Wang Z Wang PW Haskova Z. Spectral-domain OCT predictors of visual outcomes after ranibizumab treatment for macular edema resulting from retinal vein occlusion. Ophthalmol Retina (2020) 4(1):67–76. doi: 10.1016/j.oret.2019.08.009 31669329 PMC6944743

[18] Kim JH Kang SW Ha HS Kim SJ Kim JR. Overestimation of subfoveal choroidal thickness by measurement based on horizontally compressed optical coherence tomography images. Graefes Arch Clin Exp Ophthalmol (2013) 251(4):1091–6. doi: 10.1007/s00417-012-2147-9 22948949

[19] Tekin K Inanc M Kurnaz E Bayramoglu E Aydemir E Koc M. Quantitative evaluation of early retinal changes in children with type 1 diabetes mellitus without retinopathy. Clin Exp Optometry (2018) 101(5):680–5. doi: 10.1111/cxo.12667 29488254

[20] Querques G Lattanzio R Querques L Triolo G Cascavilla ML Cavallero E. Impact of intravitreal dexamethasone implant (Ozurdex) on macular morphology and function. RETINA (2014) 34(2):330–41. doi: 10.1097/IAE.0b013e31829f7495 23945638

[21] Shah AR Williams S Baumal CR Rosner B Duker JS Seddon JM. Predictors of response to intravitreal anti-vascular endothelial growth factor treatment of age-related macular degeneration. Am J Ophthalmol (2016) 163:154–66.e8. doi: 10.1016/j.ajo.2015.11.033 26705092 PMC6609150

[22] Kim M Park YG Jeon SH Choi SY Roh YJ. The efficacy of selective retina therapy for diabetic macular edema based on pretreatment central foveal thickness. Lasers Med Sci (2020) 35(8):1781–90. doi: 10.1007/s10103-020-02984-6 32095921

[23] Sandberg MA Pearce EN Harper S Weigel-DiFranco C Hart L Rosner B. The relationship of central foveal thickness to urinary iodine concentration in retinitis pigmentosa with or without cystoid macular edema. JAMA Ophthalmol (2014) 132(10):1209–14. doi: 10.1001/jamaophthalmol.2014.1726 PMC4192011 24993773

[24] Lawali D Wu G Guo Y Lin Z Wu Q Amza A. Measurement of foveal retinal thickness in myopic patients using different display modes on optical coherence tomography: A retrospective, cross-sectional study. Ophthalmol Ther (2023) 12(1):167–78. doi: 10.1007/s40123-022-00584-x PMC9834478 36289147

[25] Cho AR Choi YJ Kim YT. Influence of choroidal thickness on subfoveal choroidal thickness measurement repeatability using enhanced depth imaging optical coherence tomography. Eye (Lond) (2014) 28(10):1151–60. doi: 10.1038/eye.2014.197 PMC4194346 25214002

[26] Verma G. Standardization of cone penetrometer test method. Int J Eng Advanced Technol (IJEAT) (2018).

[27] Kawasaki R UM Ueno S. Repeatability of manual central foveal thickness measurement using optical coherence tomography. Jpn J Ophthalmol (2006). doi: 10.1007/s10384-006-0223-2

[28] Ruia S Saxena S Gemmy Cheung CM Gilhotra JS Lai TYY. Spectral domain optical coherence tomography features and classification systems for diabetic macular edema: A review. Asia-Pacific J Ophthalmology (2016) 5(5):360–7. doi: 10.1097/apo.0000000000000218 27632028

[29] Koleva-Georgieva DN Sivkova NP. Types of diabetic macular edema assessed by optical coherence tomography. Folia Med (Plovdiv) (2008) 50(3):30–8. 19009748

[30] Markan A Agarwal A Arora A Bazgain K Rana V Gupta V. Novel imaging biomarkers in diabetic retinopathy and diabetic macular edema. Ther Adv Ophthalmology (2020) 12:2515841420950513. doi: 10.1177/2515841420950513 PMC7475787 32954207

[31] Helmy YM Atta Allah HR. Optical coherence tomography classification of diabetic cystoid macular edema. Clin Ophthalmol (2013) 7:1731–7. doi: 10.2147/opth.S47987 PMC3770711 24039393

[32] Sahin M Cingü AK Gözüm N. Evaluation of cystoid macular edema using optical coherence tomography and fundus autofluorescence after uncomplicated phacoemulsification surgery. J Ophthalmol (2013) 2013:376013. doi: 10.1155/2013/376013 23738050 PMC3657399

[33] Ozdek SC Erdinç MA Gürelik G Aydin B Bahçeci U Hasanreisoğlu B. Optical coherence tomographic assessment of diabetic macular edema: comparison with fluorescein angiographic and clinical findings. Ophthalmologica (2005) 219(2):86–92. doi: 10.1159/000083266 15802932

[34] Yalçın G Özdek Ş Baran Aksakal FN. Defining cystoid macular degeneration in diabetic macular edema: An OCT-based single-center study. Turk J Ophthalmol (2019) 49(6):315–22. doi: 10.4274/tjo.galenos.2019.22687 PMC6961082 31893586

[35] Antcliff RJ Stanford MR Chauhan DS Graham EM Spalton DJ Shilling JS. Comparison between optical coherence tomography and fundus fluorescein angiography for the detection of cystoid macular edema in patients with uveitis. Ophthalmology (2000) 107(3):593–9. doi: 10.1016/S0161-6420(99)00087-1 10711901

